# Bibliographical Notices

**Published:** 1853-02

**Authors:** 


### BIBLIOGRAPHICAL NOTICES.

*Report of the Select Committee of the House of Representatives to whom was referred the memorial of* Dr. Wm. T. G. Morton, *asking remuneration from Congress for the discovery of the anæsthetic or pain-subduing properties of Sulphuric Ether.* Dr. Wm. H. Bissell, of Illinois, Chairman.

This is the report agreed upon by the committee above named, and which is now published, in advance of its presentation, with the attest of the Clerk of the House, dated June 28, 1852. It fully recognizes and endorses the claim of Dr. Morton, and is accompanied by an act granting to him the sum of $100,000, on condition that he surrenders all right and title to the patent heretofore issued to him. The report is able and learned, covering the whole field of controversy between Dr. Morton on the one hand, and Drs. Jackson and Wells on the other. In its general conclusions we are disposed to coincide. It fully makes out the case of Dr. M. as against his rival claimants, placing Dr. Jackson in the unenviable position of claiming the credit of an improvement which he certainly did not make, and in the responsibility of which he seemed by no means anxious to be involved at the time. We cannot add, however, that it so fully makes out Dr. M.'s case in his own behalf. We are willing to give him all the credit he really deserves, and we have no objection that Congress shall allow him the sum named. But we cannot agree that Dr. M. has made altogether such a discovery as the Committee report, or that to him *alone* is due the credit of introducing anæsthesia in surgery.

It does not appear, even by the showing of this report, that Dr. M. did in reality *discover* the pain-subduing power of ether, as stated in his memorial. That was pretty well known before, as the facts cited by the committee sufficiently prove. Dr. M. confesses that it was from Dr. Jackson that he learned the local anæsthetic effect of ether as applied to a painful tooth,—an observation by no means original with Dr. J., although he seems to have thought so. Many similar applications of it had been suggested. Among others that seem to be overlooked, Mr. Hugh

Neill of Liverpool had previously recommended the injection of vapor of ether into the Eustachian tube in painful affections of the internal ear, with a distinct recognition of its local anodyne power. Neither was the idea of producing general anæsthesia by inhalation of narcotic vapor original with Dr. M. He had witnessed the unsuccessful attempts of his former partner, Dr. Horace Wells, to produce it by nitrous oxide gas. The transition to sulphuric ether was obvious. The merest tyro in chemistry has, for these many years, known how similar are the effects of vapor of ether and nitrous oxide when inhaled. Both had been taken thousands of times to the extent of intoxication, and the result seen to be almost identical. Thus it appears that Dr. Marcy, of Hartford, suggested to Dr. Wells in 1844, to substitute the ether in his experiments, which he did in one instance with entire success. Dr. Morton has given a long account of the steps by which he arrived at his conclusions, showing that his difficulty arose in the outset from his ignorance of facts well known to others at the time. His merit consists simply in his having first had the courage and directness to settle conclusively by a practical test what with others had been only a suggestion or proposition. For this he certainly deserves praise, and we are not disposed to withhold it from him. What he really did discover was not the anæsthetic power of ether, but the fact that it may be given with safety to the extent requisite for annulling pain in surgical operations. Were he to claim no more than this, we imagine that his right would be generally admitted at once; and the improvement is such, that we think even an ambitious man, as Dr. M. expressly states himself to be, might be satisfied with the credit arising from it.

Much of Dr. M.'s difficulty seems also to have arisen from the spirit in which he prosecuted his inquiries. Had he consulted any competent chemist frankly, he could have learned all that he needed about sulphuric ether at one interview. Had he sought the advice of any skilful physician, he could have been enlightened readily with regard to the probable effects of his proposed experiment, and the means of guarding against any ill consequences. But, instead of this, we find him, by his own statement, cautiously propounding guarded questions concerning ether to scientific persons—stealthily picking up odd scraps of information—

trembling lest others might divine his object in catecizing them—sending his "four-ounce-phial" first to one and then to another apothecary—afraid to take the means necessary to secure him good ether, and altogether displaying an extraordinary degree of suspicion as to the honesty and honor of his neighbors. No sooner had the surgeons of Boston used the mysterious fluid which he brought with him to the Hospital, and demonstrated that it could be employed with safety, than he commenced steps for procuring a patent. The fluid itself was called Letheon, and the style of its announcement was such as to lead the profession to believe that Dr. M. had discovered *a new substance* with these extraordinary powers. Let any one recur to the journals of the day, and he cannot resist the conviction that such impression was produced *intentionally*. Now, in this business, as well as in that of the patent, there was a degree of quackery which has always rendered us indifferent as to whether Dr. M. succeeded in establishing his claim or not. It is true, his patent has turned out valueless. As Mr. Borland remarked in the debate upon this subject in the Senate, August 28th, 1852, "his patent, so far as the legal remedy extends, is worthless to him, although he has the legal right." And why worthless? Simply because the claim of exclusive right in such a case is absurd on the face of it. Mr. Smith, of Connecticut, took the true position, when he argued in the Senate that "this was not a patentable discovery." We can readily understand the issue of a patent for any new instrument or any process of art. But a patent for the exclusive use of any particular property of a given substance is beyond our comprehension. Suppose the first individual who discovered that a glass rod was rendered electric by rubbing with silk, had taken out a patent for the exclusive use of electricity. Would the deputy-marshal of the United States be called upon to apprehend and lead before the magistrate every man who rubbed a glass rod with a silk-handkerchief and therewith attracted bits of paper? If Dr. Franklin had taken out letters patent for the exclusive right to attract lightning from the clouds—supposing such an event possible,—would the authorities be obliged to scour the commons and confiscate every boy's kite? The philosopher might have patented his lightning-rod, undoubtedly, but if another investigator of nature had discovered another and better method of protecting buildings from lightning, the patent would become of no effect. The cases are parallel. Dr. Morton might have patented any particular method of inhaling ether, but the right of any man to inhale it he could not touch. Suppose we reach up to our shelf, and, taking down the ether bottle, inhale a drachm or two of it—are we liable to an action at law for the offence? The thing is so totally absurd, that it defeats itself at once. Dr. M. was aware of this at the outset, and hence the mystification about "Letheon" which could not be kept up. We are sorry to be thus hard upon him, but we think he deserves it all.

We must also be permitted to observe, that this is the most curious Congressional document that has ever fallen under our notice. If it is gotten up by the committee and published at the public expense, Dr. M. may congratulate himself upon having secured enthusiastic friends at head-quarters. In addition to the report, we have numerous documents corroborative of Dr. M.'s claim, with lithographic copies of the certificate in his favor by physicians of Boston, and of the Monthyon medal given him by the French Institute. In the body of the report we have printed copies of his tickets in the Medical School of Harvard University, and also of a diploma (honorary?) from a college to the south of this latitude, which is without a date, and has the additional peculiarity that one of its professors, (writing himself A. M.,) signs and duplicates his signature as *Georgium.* This sort of publication may be all very right and proper, but it strikes us as an immense piece of *Barnumism*, similar to that upon which we have already animadverted. We cannot resist the conviction that this pamphlet has been got up by some of the patent-agents and "claim-brokers" who now abound in Washington, benevolently ready to assist—for a consideration—any one anxious to have a finger in the long purse of Uncle Sam.

*Principles of Human Physiology, with their chief applications to Psychology, Pathology, Therapeutics, Hygiene and Forensic Medicine.* By WILLIAM B. CARPENTER, M. D., F. R. S., F. G. S. *Fifth American, from the Fourth and Enlarged London Edition, with Three Hundred and Fourteen Illustrations.* Edited with additions, by FRANCIS GURNEY SMITH, M. D., Professor of the Institutes of Medicine in the Medical Department of Pennsylvania College, etc. Philadelphia: Lea & Blanchard, 1853. pp. 1091.

The popularity of Dr. Carpenter's "Human Physiology" in this country has been strikingly attested by the numerous editions which it has gone through. Not only every English edition has been reproduced, but in one instance a second American of one of the English editions was called for. The fourth and last English edition was completed only two months since in London, and we are indebted to the enterprising American publishers for its almost simultaneous appearance in the United States.

This edition is essentially a new treatise. The author has not only remodelled his work, with a view to the modifications introduced into the details of Physiological Science, since his last edition; but he has, in many important particulars, condensed, amplified, rearranged, and altered his original views upon various topics, which "the progressive maturation of his own opinions, and his increased experience as a teacher, have caused him to look upon in a light very different from that under which he had previously regarded them."

Without attempting a complete outline of the changes interwoven by Dr. Carpenter, in the "reconstruction" of his work, we may notice some of the more striking and prominent. Perhaps of these the most important is the introduction of the *second chapter* of this edition, which comprises a general view of the "Chemical Components of the Human Body, and the changes which they undergo within it." The scope of this chapter embraces many recent discoveries, and includes perhaps more of the topics which give Physiology its varying aspect, than any other department of it. Dr. Carpenter has succeeded admirably in presenting a "faithful and concise exposition of the present state of our knowledge on this important subject." We shall especially advert only to his views on two very interesting points in this connection—"the respective relations of fibrin and albumen to the nutritive processes, and of the former to the gelatinous tissues"—and "the discoveries of M. Cl. Bernard in regard to the elaboration of sugar and fat in the liver."

The doctrines of Zimmermann and Simon, that fibrin is one of the elements of the blood "which have arisen in it from its own decay, or has reverted to it from the *waste* of the tissues," is rejected by Dr. Carpenter,

"as completely opposed by the whole physiological history of Fibrin, and more particularly by the gradual development of this ingredient in chyle, during its onward progress to the sanguiferous system; whilst, again, it seems to be entirely negatived by a comparison of the condition of fibrin with that of the known products of the disintegration of the tissues, such as urea or creatin, in which we see a marked tendency to the reproduction of purely physical or chemical conditions, (and this pre-eminently in the crystalline aggregation,) to the exclusion of those of vitality."

Fibrin, on the contrary, he believes, according to the commonly received views, to be endowed with vital force during the *assimilation* of the crude material furnished by albumen or casein, and to be "the first step in its conversion into living tissue." But, he adopts the surmise—

"that the peculiar vital powers, with which fibrin is endowed, give a special tendency to development into tissues of the fibro-gelatinous type, which may thus be almost said to be *pre-formed* in the blood; whilst the tissues of the cellulo-albuminous type *develop themselves* at the expense of some other element of the blood, possibly the globulin of the floating corpuscles."

As regards the elaboration of fat and sugar by the liver, Dr. Carpenter considers it to be the result of the disintegration of the tissues, which, in the descending process,

"resolve themselves into two classes of substances; on the one hand, saccharine, oleaginous, and resinous matters, analogous to those of plants in which carbon predominates; on the other, a set of compounds peculiar to animals, of which nitrogen is the characteristic ingredient. * * * * * The *sugar* generated by the agency of the liver, from the products of the waste or disintegration of the system that are contained in the blood, seems to be at once employed in supporting the combustive process by which the animal heat is maintained."

The chapter on "the Structual Elements of the Human Body, and the Vital Actions which they exhibit"—which includes the general doctrines of Cell-formation and of Vital Force—is also new.

The chapter on the "Physical Characters, Chemical Composition and Vital Properties of the Blood" has been greatly extended and almost entirely re-written.

"The series of chapters on the several organic functions remain the same as in the previous edition; but important additions and corrections have been made in every one."

"It is in the chapter devoted to the Functions of the Nervous System, which constitutes one-fifth of the entire volume, that the greatest additions and alterations will be found. This subject, in its Psychological as well as in its Physiological relations, has occupied more of the Author's attention than any other department of Physiology; and he now offers the more matured points of his enquiries and reflections, with some confidence, that even if his views should hereafter require modification as to details, they will be found to be fundamentally correct, and to furnish materials of some value in Psychological inquiry, as well as in the study of Mental Pathology."—*Preface*, pp. 13, 14.

We have thus adverted to some of the leading "additions and alterations," which have been introduced by the author into this edition of his Physiology. They will be found, however, very far to exceed the ordinary limits of a new edition—"the old materials having been incorporated with the new, rather than the new with the old." It now certainly presents the most complete treatise on the subject within the reach of the American reader; and while, for availability as a text-book, we may perhaps regret its growth in bulk, we are sure that the student of physiology will feel the impossibility of presenting a thorough digest of the facts of the science within a more limited compass.

It is to be observed, that a large number of *references* have been incorporated with this edition; and "to this addition, no small portion of the augmented bulk of the volume is due."

The duties of the American editor have been performed with excellent taste and discretion. His additions have been limited to an "occasional illustration of the text," and a notice of such recent discoveries as have appeared in the journals, while the sheets passed through the press. The additional matter, though not overloading the text, will be found every where appropriate and valuable.

*On Animal Chemistry in its application to Stomach and Renal Diseases.* By H. Bence Jones, M. D., etc., London. John Churchill, 1852.

We have here a London copy of this excellent work, kindly furnished by M. Bailliere of New York. It does not purport to be a complete treatise upon the subjects mentioned in the title, but is composed of twelve separate lectures, illustrating some few of the varied diseases affecting the Stomach and Renal system.

The author in the commencement pays a merited tribute to Dr. Prout, whose well known work is admitted to be the "fons et origo" of most of the treatises on the affections which Dr. Jones now discusses.

The 1st Lecture is devoted to the consideration of Food, comprising all that is new and valuable under that head, and its relation to Air, Exercise, Respiration, &c. The 2d Lecture, on Digestion, contains much information on that subject, of value. From this to Lecture 3d the transition is easy. It considers the Blood, and furnishes all that is essential upon that important component of the animal economy. Lecture 4th commences the consideration of the Urine, beginning with Calculi. Lectures 5th and 6th consider respectively the quantity and Acidity of the Urine and Uric acid. These lectures demand more than a passing notice, as they are important in a chemical point of view. Dr. Jones does not believe the statement of Prout, that the acidity of the urine is dependent upon acid urate of ammonia, yet in confirmation of Prout's views we may mention that vol. li. of the Annalen der Chemie gives two compounds—lithate of ammonia and super lithate of ammonia. Dr. Jones, however, says, "the conclusions I have arrived at are that uric acid is combined with ammonia, but the urate of ammonia is modified in form and in solubility by common salt and other saline substances which exist with it in the urine." And again, "the so-called super lithate of ammonia, I consider to be a mixture of uric acid and urate of ammonia." As it is, farther observations are necessary to determine the existence of one or two compounds, and we do not arrogate to ourselves the power of determining the true state of the question.

Lecture 7th considers Oxalate of Lime and Sulphates. Dr.

Jones here introduces to the profession a new term, "sulphuric diathesis."

He promises, that at a future time he will notice more fully the variations of the sulphates in disease, and hints that there is a peculiar class of diseases in which the sulphates are in excess.

Lectures 8th and 9th consider the Alkalescence of the Urine. Lecture 10th, Albuminous Urine. Lecture 11th, Diabetes and Diuresis; and Lecture 12th, Relation of Urine to food and animal system and methods of examination.

The work is written in a free and off-hand style, and in a manner that evinces a thorough study of the subject before presenting it to the profession. In the rage for new things we too often pass by that which may appear trivial and unimportant in order to observe the grandeur of the new.

In the volume before us attention to this fact will help us to deduce from the observations of the writer, much of value to the practitioner. It has often occurred to us, that these are the only works where the connexions of physiology and pathology are demonstrated; very frequently it happens that in pursuit of physiological knowledge we are apt to lose sight of its application to pathology, or rather forget to make the application.

The book may be placed along with the manuals of Bird, Griffith, Warwick, &c.

It comprises 139 pages, and is plainly printed and neatly bound. To the student of animal chemistry it opens a wide field for observation, and one from which he can glean much. We have no doubt that an American edition would be hailed with pleasure. The increasing attractions this subject presents to the profession, have engendered a proportional demand for information, and this volume appears opportunely to supply the want.

It is well to have such books as these in one's library. To most, the works of Bright, Prout, &c., are inaccessible; and in the 139 pages, Jones has condensed much that is eminently practical and valuable, in his own enthusiastic manner. Space does not permit us to attempt a thorough analysis of the whole work, and we must therefore close this hasty sketch with a hearty commendation of it to all who are interested in this department of medicine.

*First Principles of Chemistry for the use of Colleges and Schools.* By BENJ. SILLIMAN, Jr., M.A., M.D., &c. *With Four Hundred and Twenty-five Illustrations. Twenty-fifth Edition. Re-written and enlarged.* Philadelphia: H. C. Peck and Theo. Bliss. 1852.

We have before us a copy of the twenty-fifth edition of this excellent work, and if any evidence of its value were needed, this fact alone would be sufficient at once to mark its popularity. The whole work has been re-written and to a great extent re-arranged, according to the standard facts and doctrines now received in chemical science. The work is divided into four parts. Part I. is devoted to the consideration of PHYSICS, embracing all that is usually taught in that connexion with chemistry, as Matter, Heat, Light, &c. Part II. considers CHEMICAL PHILOSOPHY, embracing the Laws of Combination, Nomenclature, Symbols, &c. Part III. is devoted to INORGANIC CHEMISTRY, treating of the Classification of the Elements, and the consideration of each in particular, with their combinations, &c. And "last, but not least," Part IV. comprehends ORGANIC CHEMISTRY, giving under that caption the Nature of Organic Bodies, the Vegetable Acids and Alkaloids, the Blood, the Nutrition of Animals, Plants, &c. &c.

The work by its title claims to be the "First Principles of Chemistry," and it has certainly attained that mark. It is in its style clear and perspicuous, and in its matter elaborate without becoming abstruse. It is admirably adapted to the wants of the beginner in chemical research, and on this ground claims the attention of the medical teacher as a class book in the office for students. In our opinion, it is eminently suited for this purpose—presenting to the tyro a book from which he can gather all that is necessary in this go-ahead age for raising the foundations upon which to build in the future, while it will furnish to the advanced student much important information in a form convenient for ready reference. We say this after having examined the work minutely, and finding much in it to awaken the mind and refresh the memory. It is already the "Text-Book at Yale College," and several similar institutions, where it has been adopted for the use of those who consider that chemistry should be a component part of every liberal education.

The work consists of 544 pages (exclusive of the Index), of which number about 120 are devoted to the consideration of Physics, 17 to Chemical Philosophy, 200 to Inorganic Chemistry, and about 200 more to Organic Chemistry. In the latter part of the work considerable tact and care are evinced in the classification of the great mass of facts that have accumulated within the last few years in this department of chemistry. In fact, Organic Chemistry has of late attracted so much attention, although even yet not as much as is commensurate with its importance, that a chemical work without a full exposition of it is not now complete. This department has been arranged under the supervision of Mr. T. S. Hunt, who has proved himself fully competent to the task. Emanating, however, as the major part of the work does, from the pen of a "Silliman," is the best evidence of its correctness and sound chemical reasoning. The book is copiously illustrated, and it has seldom been our pleasure to observe more accurate and beautiful wood-cuts. In its typography it will vie with many other works of more pretension, and in its general "getting up" it does a deal of credit to its publishers. We wish it an extended sale and a careful perusal.

---

*Notice of the Life and Professional Services of William R. Grant, M. D.* By HENRY S. PATTERSON, M. D., Professor of Materia Medica and Therapeutics, in the Medical Department of Pennsylvania College.

Dr. Patterson has presented a highly appropriate and interesting memoir of his late colleague, who, during a short professional career, and comparatively brief sojourn here, worked his way to no little distinction and deservedly high personal estimation.

Dr. Grant was a native of Nova Scotia, whence he emigrated to Philadelphia, in the year 1836, to matriculate as a pupil of Jefferson Medical College, where he graduated in 1839. "He was immediately appointed Demonstrator of Anatomy, and Curator of the Museum of Jefferson College, which situation he filled with credit to himself and advantage to his alma mater for three years." In 1843, upon the dissolution of the original Faculty of Pennsylvania College, Dr. Grant formed one of an association which assumed the task of re-organizing the School; and his duties in this sphere, as Professor of Anatomy, were continued till his death, last Spring. As a teacher of Anatomy, he earned considerable reputation and popularity; and his labors were no doubt efficient in elevating the School to its present point of success. "In his professional relations, Dr. Grant was characterized by great amiability of temper, courteous deportment, and the strictest integrity."

---

*General Pathology, as Conducive to the Establishment of Rational Principles for the Diagnosis and Treatment of Disease; a Course of Lectures delivered at St. Thomas' Hospital during the Summer Session of* 1850. By JOHN SIMON, F. R. S., one of the Surgical Staff of that Hospital, &c. &c. Philadelphia: Blanchard & Lea; 1852.

This is the title of a new work recently re-published by Messrs. Blanchard & Lea, of this city. It consists of a series of highly interesting and instructive lectures delivered at St. Thomas' Hospital, London. Some of the new and apparently heterodox ideas therein advanced upon Pathological subjects, at first tempted us to cast aside the book, as one subverting doctrines hitherto taught and received; but when we examined the sound and cogent arguments, and beheld the unmistakeable array of evidence adduced by Mr. S. in support of his propositions, we were forced to the conclusion, that many of our long accredited and cherished opinions on the subject of disease could no longer be sustained by reason or supported by fact.

His views are plainly and concisely stated, and in such an attractive manner, as to enchain the attention of the reader; and should they be adopted by the profession at large, are calculated to produce important changes in medicine. Physicians and students will obtain from its perusal not only the latest discoveries in Pathology, but that which is even more valuable, a systematic outline for the prosecution of their future studies and investigations. Altogether, we look upon it as one of the most satisfactory and rational treatises upon that branch now extant.

